# Phylogenetically and metabolically diverse autotrophs in the world’s deepest blue hole

**DOI:** 10.1038/s43705-023-00327-4

**Published:** 2023-11-14

**Authors:** Xing Chen, Jiwen Liu, Xiao-Yu Zhu, Chun-Xu Xue, Peng Yao, Liang Fu, Zuosheng Yang, Kai Sun, Min Yu, Xiaolei Wang, Xiao-Hua Zhang

**Affiliations:** 1 Frontiers Science Center for Deep Ocean Multispheres and Earth System, and College of Marine Life Sciences, Ocean University of China, Qingdao, 266003 China; 2 Laboratory for Marine Ecology and Environmental Science, Laoshan Laboratory, Qingdao, 266237 China; 3 Institute of Evolution & Marine Biodiversity, Ocean University of China, Qingdao, 266003 China; 4 Key Laboratory of Marine Chemistry Theory and Technology, Ministry of Education, Ocean University of China, Qingdao, 266100 China; 5 Sansha Track Ocean Coral Reef Conservation Research Institute, Sansha, 573199 China; 6 College of Marine Geosciences, Ocean University of China, Qingdao, 266100 China

**Keywords:** Metagenomics, Water microbiology

## Abstract

The world’s deepest yongle blue hole (YBH) is characterized by sharp dissolved oxygen (DO) gradients, and considerably low-organic-carbon and high-inorganic-carbon concentrations that may support active autotrophic communities. To understand metabolic strategies of autotrophic communities for obtaining carbon and energy spanning redox gradients, we presented finer characterizations of microbial community, metagenome and metagenome-assembled genomes (MAGs) in the YBH possessing oxic, hypoxic, essentially anoxic and completely anoxic zones vertically. Firstly, the YBH microbial composition and function shifted across the four zones, linking to different biogeochemical processes. The recovery of high-quality MAGs belonging to various uncultivated lineages reflected high novelty of the YBH microbiome. Secondly, carbon fixation processes and associated energy metabolisms varied with the vertical zones. The Calvin–Benson–Bassham (CBB) cycle was ubiquitous but differed in affiliated taxa at different zones. Various carbon fixation pathways were found in the hypoxic and essentially anoxic zones, including the 3-hyroxypropionate/4-hydroxybutyrate (3HP/4HB) cycle affiliated to *Nitrososphaeria*, and Wood-Ljungdahl (WL) pathway affiliated to *Planctomycetes*, with sulfur oxidation and dissimilatory nitrate reduction as primary energy-conserving pathways. The completely anoxic zone harbored diverse taxa (*Dehalococcoidales*, *Desulfobacterales* and *Desulfatiglandales*) utilizing the WL pathway coupled with versatile energy-conserving pathways via sulfate reduction, fermentation, CO oxidation and hydrogen metabolism. Finally, most of the WL-pathway containing taxa displayed a mixotrophic lifestyle corresponding to flexible carbon acquisition strategies. Our result showed a vertical transition of microbial lifestyle from photo-autotrophy, chemoautotrophy to mixotrophy in the YBH, enabling a better understanding of carbon fixation processes and associated biogeochemical impacts with different oxygen availability.

## Introduction

The expansion of oxygen-deficient regions worldwide is considered a critical environmental and ecological issue that affect marine biogeochemical processes [1]. Oxygen deficiency leads to habitat compression and reduced productivity of aerobic organisms, but provides conditions favoring anaerobic metabolism. The transition from oxic to anoxic conditions has a significant impact on the biogeochemistry of the ocean across different spatial scales [2]. This is mainly contributed by shifts of respiratory and energy metabolism adopted by microorganisms under different redox conditions. Thus, it is critical to explore how oxygen availability impacts microbial physiological metabolisms and associated nutrient and energy budgets.

Blue holes are karst caverns, formed by dissolution or a fracture-type collapse of carbonate rocks and submerged as sea levels rose [3]. For the past three decades, blue holes have been widely studied, which demonstrated vertical stratification of biological community and carbonate geochemistry in the water columns [3–8]. Inland anchialine blue holes, such as the Bahamas and the Yucatán Peninsula holes, are highly stratified and differ in microbial communities from other marine and freshwater systems [7]. Marine blue holes differ from anchialine blue holes as they do not have freshwater layers, and have little photosynthetic oxygen production and restricted vertical stratification. The yongle blue hole (YBH) is the deepest marine blue hole among the world, characterized by nutritionally restricted nature and great shifts in the concentration of dissolved oxygen (DO) within three hundred meters depth [2]. The rapid transition of redox condition in the YBH provides a unique and more accessible habitat for examining biochemical processes related to carbon acquisition. Previous studies have reported vertical variation of prokaryotes [9, 10] and *Vibrio* [11] communities in the YBH in response to DO gradients. He et al. [12] provided the first glimpse of the linkage between microbial community and metabolic potential based on 16 S ribosomal RNA gene amplicons and metagenomes. Thirty-one high-quality metagenome-assembled genomes (MAGs) were retrieved recently from the Gulf of Mexico blue hole, displaying extensive biochemical capabilities for sulfur and nitrogen cycling, and representing a high level of novel microbial lineages [13]. While the microbial metabolic processes are recently being investigated, those linked to the carbon cycle along the redox gradients need more comprehensive investigation, particularly the autotrophic carbon fixation, a crucial component of the carbon cycle. Reconstruction of individual genomes from the YBH can excavate representative microbial lineages adapted uniquely to the DO gradient, and provide deeper and more accurate insights into carbon and energy metabolisms.

Autotrophy is an important metabolic strategy for microorganisms and exerts an important role in biogeochemical cycles [14]. In oxygen-depleted environments, autotrophic microorganisms participate in energy metabolism with different availability of electron sinks [15, 16]. Recently, Yao et al. [2] reported that the YBH was characterized by considerably low dissolved organic carbon (DOC) and high dissolved inorganic carbon (DIC) concentrations. This unique environment may create conditions for the growth of autotrophic microbes, as is the case in nutritionally restricted deep seas with abundant chemolithoautotrophy [17, 18]. The role of carbon acquisition coupled electron sinks in shaping these autotrophic communities has not been addressed under that condition in YBH. Since inorganic carbon fixation is energetically costly [19], mixotrophic microorganisms use or switch different strategies for carbon acquisition may constitute a cost-effective strategy to survive in the deep YBH.

In this study, we investigated the samples collected from the YBH that spanned oxygenated, hypoxic and anoxic waters. 16 S rRNA amplicons and metagenomic analyses were used to determine the finer microbial distribution patterns, carbon fixation pathways and coupled energy conservation along the redox gradients. We identified high-quality MAGs that encoded various carbon fixation pathways. These MAGs were interrogated for metabolic capabilities involved in energy generation and the potential for mixotrophic carbon utilization. Our work aimed to provide insights into the YBH carbon acquisition strategies, and revealed that metabolically flexible mixotrophic lifestyles are prevalent for microorganisms in deep YBH system.

## Materials and methods

### Sampling

The YBH is located in the largest yongle atoll of the western Xisha island which are distributed at the transition zone between outer edge of continental shelf and abyssal basin of the South China Sea (SCS, 111.768°N, 16.525°E) [2]. Water samples were collected in October 24-25, 2019 using Niskin samplers (8 L, Keruiou, Tianjin) deployed via a winch mounted on the platform from the YBH on the outer reef slope of the yongle atoll as described by Sun et al. [20]. The sampling spanned 0 to 190 m at depth intervals of 5 or 10 m. Water samples were analyzed for DO, H_2_S, salinity, temperature, NH_4_^+^, NO_2_^−^, NO_3_^−^, PO_4_^3-^, SO_4_^2−^, and DOC, following standard procedures as previous described [2, 21]. DO was fixed by adding the reagents (MnCl_2_ and NaOH/NaI) to seawater, following standard procedures [22]. Samples for H_2_S analysis were stored by adding the procedural reagents (a mixture of N, N dimethyl-p-phenylenediamine sulfate and ferric reagents) [23]. Temperature and salinity were determined on site immediately after collection using CTD. The dissolved inorganic nutrients such as NH_4_^+^, NO_2_^−^, NO_3_^−^, PO_4_^3-^, and SO_4_^2−^ were analyzed according to methods by Chen et al. [2, 21]. Nutrients were detected by standard colorimetric method on AA3 continuous flow analyzer (Seal Analytical Ltd., UK). Seawater samples were filtered sequentially through 3 μm (TSTP, 142 mm, Millipore, Burlington, MA, USA) and 0.22 μm (GTTP, 142 mm, Millipore, MA, USA) polycarbonate membranes. Hence, the microbial communities collected on the 3 μm and 0.22 μm filters were designated as particle-associated (PA) and free-living fractions (FL), respectively. Each sample was named according to its fraction size and sampling depth. In total, 57 samples from seven different water depths, including 29 free-living samples and 28 particle-associated samples. All filters were stored in liquid nitrogen onboard, and at −80 °C in the laboratory for DNA extraction.

### DNA extraction, sequencing and quantitative PCR

Total DNA and RNA for amplicon sequencing were extracted from the 3 μm and 0.22 μm membranes by the DNeasy PowerSoil Pro Kit (Qiagen, Germany) and RNeasy PowerSoil Total RNA Kit (Qiagen, Germany) respectively, according to the method described in Liu et al. [24]. The DNA and RNA samples were sequenced by Majorbio Bio-pharm Technology Co., Ltd. (Shanghai, China). Primers 515 F (5′-GTGYCAGCMGCCGCGGTAA-3′) and 806 R (5′-GGACTACNVGGGTWTCTAAT-3′) were used to amplify the V4 region of the 16 S rRNA gene. Amplification was performed in a reaction system containing 2 µl of 10×polymerase chain reaction (PCR) buffer, 2 µl of 2.5 mM dNTPs, 0.5 µl of each primer (5 µM), 0.2 µl of rTaq polymerase, 0.2 µl of bovine serum albumin and 10 ng of template DNA. The PCR reaction was 95 °C for 3 min, 25 cycles of 95 °C for 30 s, 55 °C for 30 s, 72 °C for 30 s and a final extension at 72 °C for 5 min. After that, the purified PCR amplicons were sequenced on Illumina Miseq PE300 (MiSeq Reagent Kit v3) platform. The 16 S rRNA raw data were trimmed the barcodes and primers, and filtered low-quality reads for subsequent analyses. Reads that shorter than 50 base pairs with an average quality score lower than 20 and with any ambiguous bases, were filtered. Then, the clean reads were clustered into operational taxonomic units (OTUs) with a similarity cutoff of 97% by VSEARCH [25], and classified by the RDP classifier against the SILVA v138 [26]. Fourteen metagenomic samples from seven different water depths (0, 30, 50, 90, 120, 140 and 170 m) were extracted (each greater than 1.5 μg), and were sent to BGI (Shenzhen, China) for metagenomic sequencing. DNA fragment library (300-400 bp) was constructed by the BGI sequencing platform.

The abundance of bacterial and archaeal 16 S rRNA genes was quantified by quantitative PCR (qPCR) with primers Bac-967F/Bac-1046R and Arc-967F/Arc-1060R, respectively. Each 20 μl reaction system contained 10 μl of SYBR Green real-time PCR Master Mix (TaKaRa, Tokyo, Japan), 0.4 μl of ROX reference dye (TaKaRa, Tokyo, Japan), 0.2 μM concentrations for each forward and reverse primer, 2 μl of template DNA, and double-distilled water. The qPCR cycle included activation step at 94 °C for 3 min, 35 cycles of a three-step reactions involving denaturation at 94 °C for 30 s, annealing at 57 °C for 45 s, and extension at 72 °C for 30 s. Standard curves for each qPCR assay were obtained by the amplification of plasmids containing the corresponding PCR products of each 16 S rRNA gene primer pair.

### Metagenomic assembly and binning

Metagenomic raw reads were quality-controlled through clipping off primers and adapters, filtering low-quality reads (quality scores <20) and trimming by Trimmomatic v3.6 [27]. The clean reads from each sample were assembled separately using MegaHit version 1.1.2 after filtering [28]. Binning of metagenome was performed using MetaBAT v2.14 [29] and MaxBin v2.2.7 [30], which were then merged and refined with MetaWRAP v1.2 [31]. Metagenomic reads were remapped to each MAG and then reassembled by SPAdes [32] in careful mode to further improve the quality of the MAGs. Subsequently, the completion and contamination of MAGs was further examined by CheckM version 1.0.8 [33].

### Gene prediction and functional annotation

The metagenomic sequences and individual MAG were performed for gene prediction using prodigal version 2.6.3 with default settings [34]. All metagenomic samples were clustered to generate the non-redundant gene set using CD-Hit with 95% identity and 90% coverage settings [35]. The non-redundant genes were mapped against the high-quality reads of each individual metagenome by BWA-MEM to determine the relative abundance of each gene in the non-redundant gene set [36]. To assess and compare the relative abundances of specific pathways/genes in different metagenomic samples, a DiTing software (https://github.com/xuechunxu/DiTing, Version 0.5, Qingdao, Shandong, China) was used by unbiased specific formulae [37]. Metabolic pathways of MAGs were further predicted against the KEGG database using the GhostKOALA panel, and against the Pfam, TIGRfam and custom HMM databases [38]. Firstly, the marker genes of each carbon fixation pathway and other pathways for energy sources were selected. Although the marker gene can reflect the existence of each carbon fixation pathway, the integrity of pathway needs to be further verified. Thus, the completeness of specific metabolic pathways in individual MAGs was estimated by reconstruction of whole pathways using KEGG-Decoder (www.github.com/bjtully/BioData/tree/master/KEGGDecoder, Los Angeles, CA, USA). Since not all genomes containing carbon fixation pathways are strict autotrophic lifestyles, carbohydrate metabolisms were also identified in MAGs. The dbCAN web server was used for carbohydrate-active gene identification, which integrated three tools (HMMER, DIAMOND and Hotpep) [39]. The bubble diagram and heatmap were generated in the R software.

### Phylogenetic analyses of the reconstructed genomes

The taxonomy of the 223 MAGs (bins) were classified by GTDB-Tk v1.3 [40]. Phylogenetic relationships among the 202 bacterial MAGs or 21 archaeal MAGs were inferred by constructing a maximum-likelihood tree using 120 bacterial and 122 archaeal marker genes identified in GTDB-Tk. Bac120 or arc122 proteins were predicted using the GTDB-Tk identify module. Concatenated multiple sequence alignment was performed with the GTDB-Tk align module. The phylogenomic trees of MAGs with IQ-TREE version 1.6.1 under the LG + R10 and LG + R4 model were inferred [41]. The trees were annotated using the Interactive Tree of Life (iTOL) webtool for better visualization [42].

## Results and discussion

### Information of vertical stratification in the YBH

**Fig. 1:**
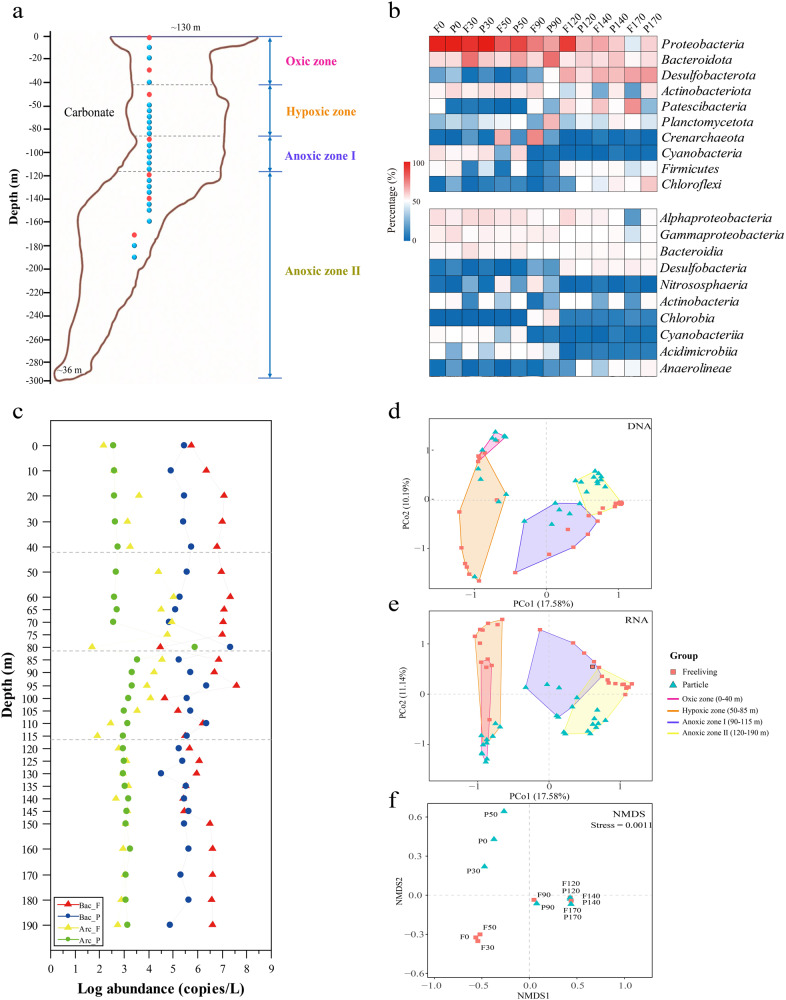
The information of sampling and microbial community composition in the YBH. **a** Vertical cross-section and sampling sites through the Sansha Yongle Blue Hole. Blue dots represent samples that were analyzed by 16 S rRNA sequencing; Red dots represent samples that were metagenomic sequenced. **b** The relative sequence abundance of dominant microbial groups (phylum and class, top 10). **c** The abundance of bacterial and archaeal 16 S rRNA genes for free-living and particle-associated lifestyles by qPCR analysis. F, free-living lifestyle. P, particle-associated lifestyle. **d**, **e** The PcoA results about DNA and RNA sequencing of microbial present across fine depths. **f** Nonmetric multidimensional scaling (NMDS) of the microbial communities based on the functional compositional similarity (Bray–Curtis distances) among the 14 samples based on clusters of KEGG orthologous groups (KOs).

Oxygen-depleted marine systems are natural laboratories for understanding ecosystem function under oxygen limitation across redox gradients [1]. These ecosystems represent critical resources for discovery of novel microbial diversity and unforeseen biochemical cycles [43–47]. The YBH was spatially isolated from surrounding seawaters below 10 m depth. Sharp transitions of DO were observed at 10 m, 40 m and 90 m (Fig. S1, and Table S1). With the disappearance of DO at a depth of 100 m, the concentrations of NH_4_^+^ and H_2_S increased gradually to 130 m, and then displayed a sharp increase to 150 m. Below this depth, NH_4_^+^ and H_2_S kept relative stable. There was a clear fluctuation of NO_x_^−^ concentration from 40 m to 100 m. NO_3_^−^ increased at 40 m, peaked at 60 m and 85 m before a sharp decrease, whereas NO_2_^-^ showed the highest concentration at 90 m. Salinity and temperature, which ranged from 33.65 to 34.48 ppt and 28.66 to 15.43 °C respectively, began to change significantly at 40 m. Considerably low DOC and high DIC concentrations may support abundant autotrophic communities. Based on these parameters, the YBH characterized by water column stratification and sharp DO gradients can be divided into four zones: an oxic zone (0–40 m, DO > 100 μM), a hypoxic zone (50–85 m, 20 μM < DO < 100 μM), an essentially anoxic zone (anoxic zone I; 90–115 m, DO below detection limit, NO_2_^−^ > 0, NO_3_^−^ > 0, H_2_S > 0), and a completely anoxic zone (anoxic zone II; 120–190 m, DO = 0, NO_2_^−^ = 0, NO_3_^−^ = 0) (Fig. 1a).

### Phylogenetic diversity of microbial community in the YBH

To explore the depth profile of the YBH microbial community, qPCR, 16 S rDNA and rRNA amplicon sequencing was performed (Fig. 1b, c, S2, S3, and Table S2). The qPCR analysis showed bacterial and archaeal abundance were relatively stable across water depths, except for some fluctuations that occurred from the bottom of the hypoxic zone to essentially anoxic zone (70 m to 110 m). At these depths, bacteria and archaea with different lifestyles displayed contrasting variation trends. For example, at 80 m, particle-attached microorganisms that collecting through a 3 μm membrane filtration presented the highest abundance, whereas free-living microorganisms that sequentially collecting through a 0.22 μm membrane filtration showed the opposite trend (Fig. 1c). Two distribution patterns reflected the change of sharp redox condition at 80 m. The community composition shifted at different zones, and showed preference for free-living and particle-attached lifestyles (Fig. 1b, S2, and S3). *Proteobacteria* and *Bacteroidota* were abundant in the oxic zone and essentially anoxic zone. An unexpected increase of these populations was observed in the PA fraction of completely anoxic zone. *Cyanobacteria* showed a high abundance within the oxic zone and in particle samples of the hypoxic zone. *Chlorobia*, *Planctomycetota*, and *Nitrososphaeria* comprised the main active populations at 90 m. The dominant groups in completely anoxic zone were *Desulfobacterota*, *Chloroflexi* and *Anaerolineae*. Also, *Patescibacteria* and *Cloacimonadota* showed increased abundance in the free-living factions. A principal co-ordinates analysis (PCoA) confirmed the distinctions of microbial community with different lifestyles (FL or PA) across different DO water layers (Fig. 1d, e). Furthermore, functional profiles derived from metagenomics by nonmetric multidimensional scaling (NMDS), suggested that community lifestyle (FL *verse* PA) rather than geographic origin shaped the functional structure of the microbial communities in the oxic and hypoxic zones. PCoA and NMDS analysis separated the communities with different lifestyles into four categories that matched the DO gradient. These results underscored the importance of DO in structuring autotrophic communities, in agreement with previous finding in aquatic ecosystems [48–50].

A total of 223 MAGs were retrieved from the YBH, with an estimated genome size between 0.6 and 13.9 Mbp (average 3.3 Mbp) (Table S3). More than half (119 out of 223) of the MAGs had high completeness (>90%) and low contamination levels (<5%), representing high-quality draft MAGs based on recently established standard [51]. The remaining MAGs were medium quality (>50% completeness, <10% contamination). Estimated completeness of the reconstructed genomes was mostly higher than 75%, and contamination was less than 5% as assessed by CheckM. A phylogenetic analysis of the single-copy, protein-coding marker genes (120 for bacteria and 122 for archaea) revealed 202 bacterial and 21 archaeal genomes, which were affiliated with 32 phyla, including *Proteobacteria* (36), *Bacteroidetes* (25), *Desulfobacterota* (24), *Planctomycetota* (19), *Chloroflexota* (14) and other unclassified bacteria according to the GTDB database (Fig. S4, S5, S6). It was noted that 5, 15, and 78 MAGs were unassigned taxonomically at the order, family and genus level, respectively. Phylogenomic inference revealed monophyletic clades of archaeal bin29 and bin202, and bacterial MAGs (bin207, bin24 and bin54) that comprised unknown microorganisms. These MAGs belonged to uncultivated lineages that lacked previous metabolic or phylogenetic insights, indicating high diversity and novelty of the YBH microbiome. High levels of novel microbial lineages have recently been discovered in an Amberjack blue hole in the Gulf of Mexico [13]. Higher number of high-quality autotrophic MAGs of 64 were retrieved from the YBH than other aquatic systems, e.g., 11 MAGs mainly with WL pathway in subseafloor [49] and 33 MAGs mainly with rTCA cycle in cold seep sediments [52]. These may suggest that autotrophy was a prevalent metabolic style in this unique environment.

### Carbon fixation pathways implemented by microorganisms at different water layers

**Fig. 2:**
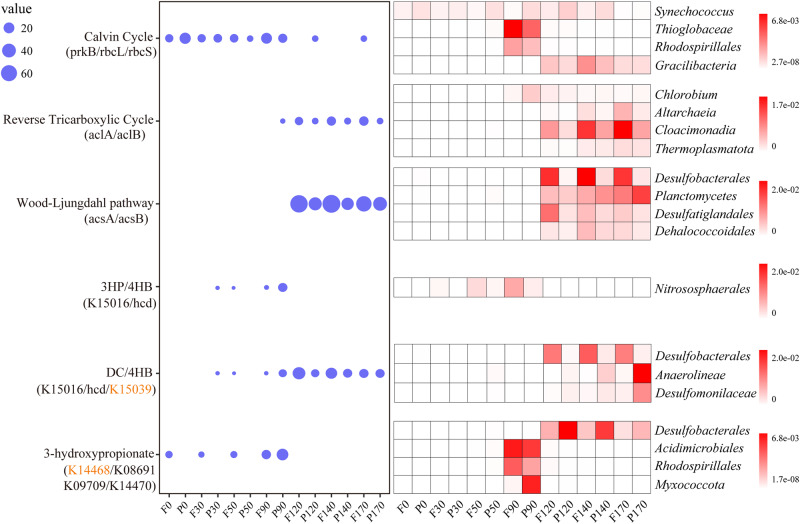
The distribution of carbon fixation pathways with depth in YBH samples. The average abundance of the carbon fixation marker genes at different depths (left). *prkB*: phosphoribulokinase, K00855; *rbcL*: ribulose-bisphosphate carboxylase large chain, K01601; rbcS: ribulose-bisphosphate carboxylase small chain, K01602; *aclA*: ATP-citrate lyase alpha-subunit, K15230; *aclB*: ATP-citrate lyase beta-subunit, K152301; *acsA*: anaerobic carbon-monoxide dehydrogenase, K00198; *acsB*: acetyl-CoA synthase, K14138; 3-hydroxyacyl-CoA dehydrogenase: K15016; 4-hydroxybutyryl-CoA dehydratase: K14534; 3-hydroxypropionate dehydrogenase: K15039; malonyl-CoA reductase: K14468; malyl-CoA/(S)-citramalyl-CoA lyase: K08691; 3-methylfumaryl-CoA hydratase: K09709; 2-methylfumaryl-CoA isomerase: K14470. The orange marked genes mean deletion in all samples. The top four affiliated groups containing them shown in the heat map (right) in depth-profiled samples determined by metagenomic analysis. F free-living, P particle-associated.

To explore the microbial metabolic strategy of inorganic carbon acquisition, we detected the distribution of marker genes (average abundance) representing different carbon fixation pathways at different depths (Fig. 2). The related genes of six previously characterized carbon fixation pathways including the Calvin–Benson–Bassham (CBB) cycle, reductive tricarboxylic acid (rTCA) cycle, Wood-Ljungdahl (WL) pathway, 3-hyroxypropionate/4-hydroxybutyrate (3HP/4HB) cycle, dicarboxylate/4-hydroxybutyrate (DC/4HB) cycle and 3-hydroxypropionate bi-cycle (3HP), were detected. Among them, genes encoding the Group I/II ribulose-1,5-bisphosphate carboxylase/oxygenase (RuBisCO) and phosphoribulokinase (*prk*), key enzymes in the CBB cycle, were ubiquitous throughout the water column, but especially dominant in the oxic and hypoxic zones. The 3HP/4HB pathway was restricted to 50 m and 90 m, and abundant in the 90 m essentially anoxic zone. In contrast, the *acsA*/*acsB* genes, encoding carbon monoxide dehydrogenase/acetyl-CoA synthase associated with the WL pathway, were restricted to completely anoxic zone. Also, the rTCA cycle was enriched in completely anoxic zone. In addition, lack of partial marker genes of the DC/4HB and 3HP cycles indicated the incompleteness of these two pathways in the YBH. Overall, the WL pathway was markedly abundant in the YBH compared with other pathways, and these findings reflected different carbon fixation processes shifting from the top oxic, hypoxic, essentially anoxic zone, to completely anoxic zone.

**Fig. 3:**
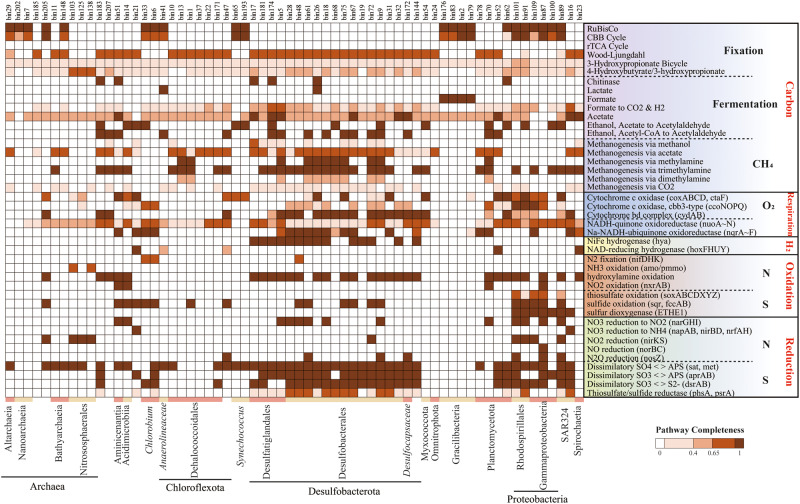
The information of carbon fixation pathways and coupled redox metabolism in autotrophic MAGs. These MAGs (top) harbor complete or near-complete carbon fixation pathways (Completeness>67%). Taxonomic classifications of MAGs are represented at bottom of heatmap by different colors. Corresponding genes involved in each pathway are shown in parentheses. Color of each cell refers to completeness of enzymes involved in each pathway.

**Fig. 4:**
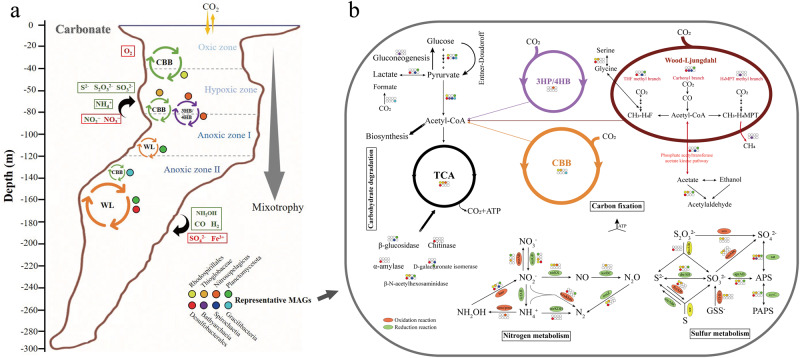
The summary of carbon fixation and energy metabolisms along vertical gradient in the world’s deepest blue hole. **a** The overview of carbon fixation pathways and representative clades along vertical gradient. Cycle circles represent distribution of different carbon fixation pathways, and the colored dots were eight representative clades nearby. The box is the possible source of electron donors and electron acceptors for carbon fixation in different layers. The big arrow reflected the tendency of mixotrophy for these clades from the top to the bottom. **b** The inferred metabolic capabilities in eight representative MAGs. Predicted metabolic pathways and corresponding genes that may drive carbon fixation in eight representative MAGs are indicated by colored dots to demonstrate the comparison between clades. Metabolic pathways included carbon fixation, sulfur, nitrogen metabolisms and carbohydrate degradation. The colored ellipse represent oxidation, reduction reaction and disproportionation. The white dots represent missing this pathway.

Next, we explored the taxonomic distributions of the carbon fixation potential (Figs. 3, 4a, and Table S4). A total of 64 MAGs possessed the complete (completeness=1) or incomplete (0.67<completeness<1) carbon fixation pathways. Genes encoding the complete RuBisCO subunits for the CBB cycle were detected in 21 of the 64 MAGs. MAGs harboring a complete CBB cycle were affiliated to aerobic phototrophs *Synechococcus* and *Alphaproteobacteria* (mainly *Rhodospirillales*) in the oxic zone. The interactions between *Synechococcus* and *Rhodospirillales* could increase aggregate formation and particle sinking [53], which could enhance the contribution of *Synechococcus* to the biological carbon pump in the YBH. With decreasing DO gradient in the intermediate layers (hypoxic zone and essentially anoxic zone), most MAGs that possessed the CBB cycle were *Gammaproteobacteria* (*Thioglobaceae* and *Arenicellales*). These results were consistent with observations in the ocean [19] showing that the CBB cycle occurred mainly in photo- and (aerobic) chemo-autotrophic Alpha-, and *Gamma-proteobacteria* in addition to *Cyanobacteria*. Beside the CBB cycle, the intermediate layers possessed various carbon fixation pathways, including the 3HB/4HP cycle (*Nitrososphaeria*) and WL pathway (*Planctomycetota*). Three archaeal MAGs affiliated to ammonia-oxidizing archaea *Nitrososphaeria* harbored a complete 3HP/4HB pathway, and displayed a narrow distribution restricted to 50 m and 90 m. We also found that the CBB cycle affiliated to *Gracilibacteria* MAGs was present in the bottom completely anoxic zone, which provided the first evidence of autotrophy in this group known to be piezophiles [54].

Compared with the pathways (CBB cycle, 3HP bicycle and 3HP/4HB) under aerobic conditions, CO_2_ fixation pathways (DC/4-HB, rTCA cycle, and the WL pathway) used by anaerobic or microaerophilic microorganisms, require significantly less energy for synthesizing a three-carbon unit from CO_2_ [19]. Thus, anaerobic autotrophs usually utilize oxygen-sensitive but energy more efficient carbon fixation pathways. In this study, almost 2/3 of the MAGs (41 out of 64) possessed the near-complete WL pathway, which were assigned taxonomically to *Planctomycetes*, *Dehalococcoidales* of *Chloroflexota*, and *Desulfobacterales* and *Desulfatiglandales* of *Desulfobacterota* in completely anoxic zone. The energy efficient WL pathway may provide an advantage for their growth under extreme anoxic conditions. The marker genes *aclA*/*aclB* involved in the rTCA cycle also occurred in anoxic zones but not detected in MAGs. The possible explanation was that the WL pathway is more energy-efficient than rTCA cycle, and key enzymes of the WL pathway are highly sensitive to oxygen [55]. The unique redox condition of nutritionally restricted YBH may meet the strict requirements of predominant microorganisms. Overall, shift of autotrophic species along the water layers suggested adaptive advantages of different carbon fixation mechanisms as a response to DO gradient. The unexpected result that DC/4HB and 3HP cycles were incomplete indicated that these pathways could be more diversified than currently thought, or that an unknown pathway using shared enzymes with these cycles could be operating in the ocean.

### Versatile energy-conserving strategies in the YBH

**Fig. 5:**
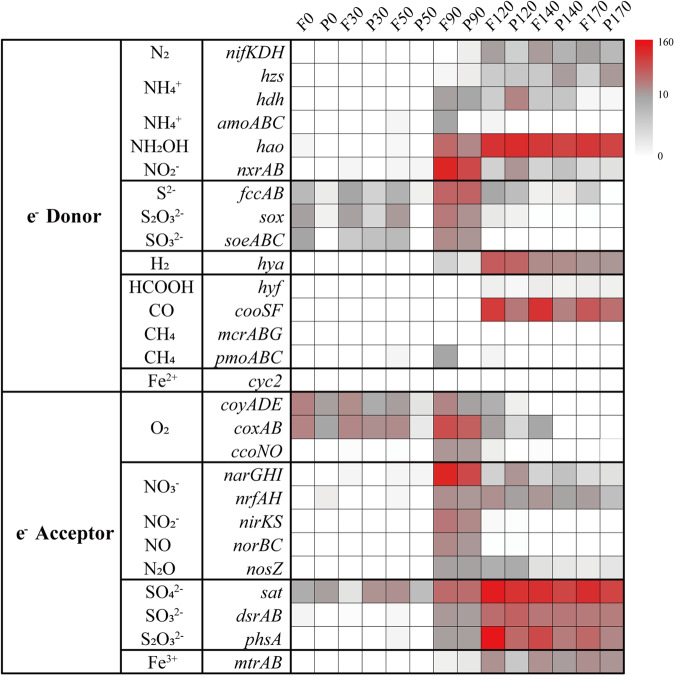
Electron donors and electron acceptors that may couple with carbon fixation within the YBH. Heatmaps showed the marker genes abundance for electron donors and electron acceptors at different depth. Pathways that were not represented in a given sample remain white.

Unique redox gradients and chemical features in the YBH, such as abrupt decline of NO_3_^−^, NO_2_^−^ and O_2_, and an increase of H_2_S and NH_4_^+^ below 100 m (Fig. S1), indicated that metabolically versatile autotrophy could couple successional redox to acquire energy for carbon fixation. We explored the potential energy metabolisms that sustained carbon fixation (Figs. 3, 4b, 5). We found the sulfur-oxidizing genes were abundant in the hypoxic zone and at 90 m, and high abundance of the *narGHI* gene corresponded to a decline of NO_3_^−^ and rise of NO_2_^−^ at 90 m essentially anoxic zone (Fig. 5, S1). Sulfur compounds are a well-characterized as electronic source for chemolithoautotrophy in the deep sea [56, 57]. The MAGs, which were identified as *Rhodospirillales* (bin101, 91) and *Thioglobaceae* (bin100, 87) that were abundant in the hypoxic zone and upper essentially anoxic zone, possessed a variety of genes conferring to use more than one type of sulfur compound, including thiosulfate oxidation (*sox*), sulfide: quinone oxidoreductase (*sqr*, *fccAB*), and sulfur dioxyenase (Fig. 3, and Table S4). Bin101 (*Rhodospirillales*) and bin87 (*Thioglobaceae*) had the potential to perform both sulfur oxidation and sulfate reduction, likely depending on oxygen concentration and/or redox potential. The chemoautotrophic growth of *Rhodospirillales* was sustained by sulfur oxidation, respiring oxygen (microaerobic growth) or N_2_O (anaerobic growth) [58]. Moreover, these groups had *narGHI* genes for dissimilatory nitrate reduction and *nosZ* gene for N_2_O reduction (Fig. 4b). The respiration processes may change significantly with gradual decline of DO, and likely shifted to dissimilatory nitrate reduction in essentially anoxic zone, with a maximum value of *narGHI* genes at 90 m (Fig. 5). Confirming prior study [59], *Nitrososphaeria* MAGs may utilize ammoxidation (*amoABC*) and 3HP/4HB cycle for carbon fixation, and nitrite (*nirKS*) is used as an electron sink (Fig. 4b). Therefore, the intermediate layers were driven by energy derived mainly from the various carbon fixation pathways and potential energy-conserving pathways. Also, the anaerobic anammox reaction occurred in the anoxic zones, with high abundance of *hzs* and *hds* genes (Fig. 5). Marine denitrification and anaerobic anammox are responsible for a significant portion of nitrogen loss from stratified anoxic zones [60, 61]. Denitrification by *Thioglobaceae* may catalyze nitrogen loss in essentially anoxic zone. Anammox by *Planctomycetota* may be the main process involved in nitrogen loss in completely anoxic zone, where nitric oxide and ammonia were oxidized to nitrogen gas (N_2_) as the final product. However, abundant *nifH* gene indicative of nitrogen fixation potential in completely anoxic zone, may account for a compensation to the nitrogen loss.

Steady accumulation of H_2_S is indicative of a strong redox gradient with sulfate reduction as a dominant respiration pathway in completely anoxic zone. Near half autotrophic MAGs retrieved from the YBH had complete pathways for dissimilatory sulfate reduction, predicting that the use of sulfate as a terminal electron acceptor is thermodynamically favorable in completely anoxic zone. *Desulfobacterales* and *Planctomycetota* MAGs may potentially utilize hydroxylamine (*hao*) and sulfate/sulfite (*sat*/*dsrAB*) as electron sink for energy-conserving, whereas the possible electron sink used by *Dehalococcoidia* MAGs was not evident (Fig. 4b). *Dehalococcoidia* may derive energy from organohalide compounds through dehalogenation [62]. Except nitrogen and sulfur metabolisms, other fermentation and redox metabolisms also played important roles in completely anoxic zone. For example, not only the complete CBB pathway was identified, but also only the metabolism for fermentative formate production was found, and without any other oxidative-reductive electron sinks in *Gracilibacteria* MAGs (Fig. 4b). Organisms belonging to *Gracilibacteria* are most probably fermentative lifestyle, lacking any type of electron transport pathway [63]. This suggested that *Gracilibacteria* may be strictly obligate autotrophs and utilize formate fermentation as an energy source for carbon fixation. Moreover, H_2_ and CO oxidation were explored as potential alternative energy sources [64]. The presence and high abundance of hydrogenases (*hya*) and anaerobic CO dehydrogenase (*cooSF*, K00196 and K00198) in completely anoxic zone (Fig. 5, and Table S5), suggested that anaerobic CO oxidation and hydrogenotrophic metabolism were important energy supplements for microorganisms in a nutritionally restricted environment. Anaerobic conversion of CO has been reported for carbon assimilation pathways in sulfate reducing bacteria [65]. The co-occurrence of CO dehydrogenase and sulfate reductase in *Desulfobacterota* MAGs (bin9, bin174 and bin181) (Fig. 3), suggested that they may potentially utilized CO oxidation and sulfate reduction allowing them to derive energy for carbon assimilation through the WL pathway. H_2_-oxidation has been described in hydrothermal vents [66] or subsurface microbial communities [67], but *Desulfatiglandales* and *Desulfobacterales* in completely anoxic zone also contained nickel-iron (Ni-Fe) hydrogenases for hydrogen metabolism, expanding the ecological niches of microbial H_2_ oxidizers (Fig. 3). In addition, the YBH is an anchialine cave system that generated minerals (e.g., Fe-bearing minerals), which may be exploited for energy. Although *cyc2* gene related to iron oxidation was not found, the enrichment of reduced Fe^3+^ gene (*mtrAB*) in completely anoxic zone suggested that chemosynthetic H_2_-based metabolisms are supported by water-rock reactions on Fe-bearing mineral surfaces. Methyl-Coenzyme M reductase (*mcr*) that catalyzes the final step of methanogenesis in methanogenic archaea and the first step in the anaerobic oxidation of methane by anaerobic methanotrophs, was not detected in any metagenomes or MAGs, suggesting that methane metabolism is likely present at a low level in the YBH. Taken together, steep environmental gradients of the YBH favored multiple types of carbon fixation, and energy generation by providing versatile electron donors and electron acceptors.

### Unique WL carbon fixation pathway in the deep YBH

**Fig. 6:**
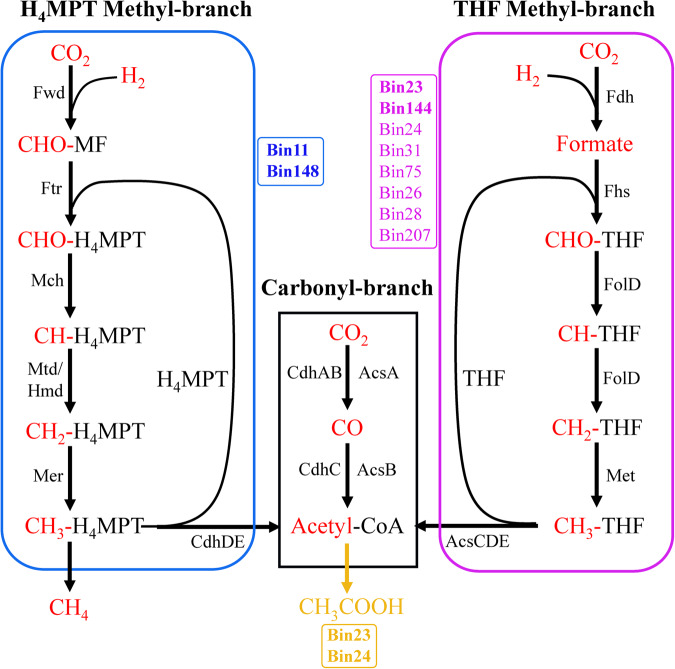
The Wood–Ljungdahl pathway summary of carbon fixation MAGs. The WL pathway consists of the carbonyl and methyl branches that reversibly reduce CO_2_ to acetyl-CoA. THF and H_4_MPT generally regarded as two versions of the methyl branch. The complete WL pathway was considered present only if all of the genes encoding at least one full enzymatic pathway capable of carrying out that metabolic process were found in genomes. These MAGs all contained the genes of carbonyl-branch. Blue and pink represent H_4_MPT and THF methyl-branch, respectively. The bold pink Bins (Bin23, Bin144) represent that harbored complete H_4_MPT methyl-branch, other regular pink Bins represent that lacked the *fdh* gene at first step but have alternative formate dehydrogenase subunits. Thus, a total of ten Bins could contain the complete WL pathway. The bold yellow Bins represent that can generate acetyl-CoA to acetic acid via the complete WL pathway and phosphate acetyltransferase-acetate kinase pathway.

The WL pathway was further clarified for completeness based on the current KEGG Carbon Fixation Pathway Modules, considering its prevalence and incompleteness of MAGs (Fig. 6). The WL pathway consists of the carbonyl and methyl branches that reversibly reduce CO_2_ to acetyl-coenzyme A (acetyl-CoA). Although certain archaea can also use tetrahydrofolate (THF) as a C1 carrier [68], THF and tetrahydromethanopterin (H_4_MPT) generally regarded as version of the methyl branch of bacteria and archaea, respectively (Fig. 6). Also, we listed other metabolic genes that may associate with the WL pathway (Table S6). We found that ten MAGs from the YBH possessed complete or near-complete WL pathway. Two MAGs (*Spirochaetia*, bin23 and *Desulfomonilales*, bin144) possessed a full suite of enzymes functioning in both branches of the WL pathway (Fig. 6). By contrast, other MAGs contained a near-complete WL pathway by missing one gene that encoded typical formate dehydrogenase (*fdh*). The *fdh* performs the first step in the methyl branch of the Wood-Ljungdahl pathway, which results in the production of formate [69, 70]. However, we identified putative *fdh* genes in some genomes that potentially utilized alternate routes of formate production by using of analogous subunits from other formate dehydrogenases (Table S6). Similarly, a total of eight MAGs may accomplish the complete WL pathway by employing alternative route (Fig. 6). Previous study of the incomplete Wood–Ljungdahl pathway in *Dehalococcoides mccartyi* indicated that this pathway was able to incorporate exogenous formate to support serine biosynthesis and cleaves acetyl-CoA to generate methyl-THF for methionine biosynthesis, serving as a unique substitute of the missing methylene-tetrahydrofolate reductase function [71]. Thus, other MAGs such as bin174 and bin68 that lacked *folD* gene may be closely related to serine metabolism. Two archaeal MAGs (*Bathyarchaeia*, bin11 and bin148) combined with the archaeal WL-H_4_MPT pathway for complete carbon fixation.

**Fig. 7:**
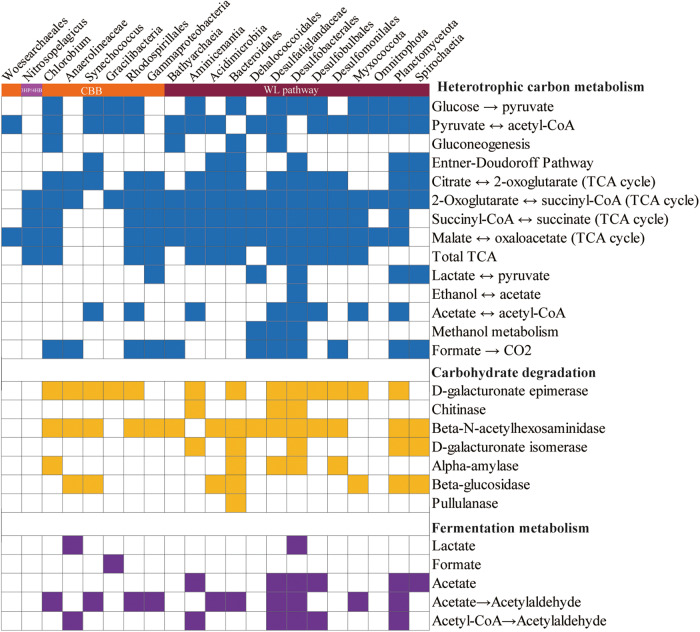
Substrate utilization overview of carbon fixation MAGs taxa. Filled boxes indicate the presence of the metabolic process or bioenergetic complex in the corresponding genome while empty boxes indicate its absence. A metabolism was considered present only if the marker genes encoding full enzymatic pathway capable of carrying out that metabolic process were found in the genome.

The WL pathway is now known to exist in a variety of forms that are used in reductive or oxidative directions in bacteria and archaea with diverse metabolic processes, usually including carbon fixation for acetyl-CoA, acetogenesis, methanogenesis, and sulfate reduction [72]. The pathway is used in reductive direction for carbon dioxide fixation and energy conservation during autotrophic growth by homoacetogenic bacteria (e.g., *Moorella thermoacetia*, *Clostridium aceticum*), hydrogenotrophic methanogens and autotrophic sulfate-reducing prokaryotes [73]. In this study, although 12 MAGs with the WL pathway had the phosphate acetyltransferase acetate kinase pathway for acetyl-CoA to acetate, only bin23 *Spirochaetia* and bin144 *Desulfomonilales* harbored the complete WL pathway (Figs. 6, 7). Bin24 (*Omnitrophota*) and bin207 (*Abyssubacteria*) among the eight MAGs with the complete WL pathway has no acetate and few fermentations metabolism, suggesting that they may use complete WL pathway for autotrophic carbon fixation (Fig. 7). The remaining bins (*Desulfobacterales*) among them as sulfate-reducing bacteria exploited the carbonyl-branch of the WL pathway in reverse to catalyze the conversion of acetate into acetyl-CoA, and generate metabolic energy by coupling sulfate reduction. A *Desulfobacterales* MAG (bin68) encoded a complete THF-WL methyl branch but lacked carbon-monoxide dehydrogenase for the carbonyl branch, suggesting that this MAG may only use tetrahydrofolate (THF) as C_1_ carrier rather than autotrophic carbon fixation. H_4_MPT methyl branch of the WL pathway is closely related to hydrogenotrophic methanogenic archaea. Only bin148 *Bathyarchaeota* that contained an *ech* (K14086-K14091), or ‘energy conserving’, hydrogenase gene (Table S5). Thus, hydrogenotrophic methanogenesis and the WL pathway may be unique and efficient energy utilization model of methanogenic archaea under energy limit YBH.

### Mixotrophic lifestyle of carbon fixing microbiomes in the YBH

**Fig. 8:**
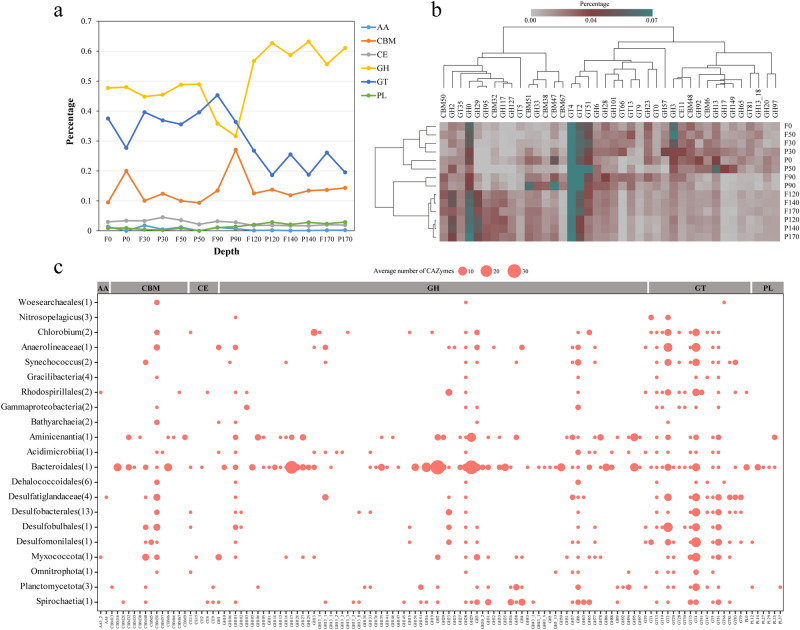
The utilization of carbohydrate-active enzymes (CAZymes) for miroorganisms in the YBH. **a** Distribution of CAZymes across different water depths in the YBH. AA auxiliary activity, CBM carbohydrate-binding module, CE carbohydrate esterase, GH glycoside hydrolase, GT glycosyltransferase, PL polysaccharide lyase. **b** The clustering of CAZymes famillies within different depth.

In addition to the capacity to fix inorganic carbon, some autotrophic microorganisms in this study are probably able to utilize a variety of organic carbon compounds (Figs. 7, 8). Analysis of CAZymes in metagenomes and MAGs revealed that these microorganisms may have large latent potential for organic carbon degradation. Glycoside hydrolases (GHs) catalyzing the hydrolysis of glycosidic linkages had the highest abundance in the YBH, and showed higher values at completely anoxic zone (Fig. 8a, and Table S7, S8). Glycosyltransferases (GTs) and carbohydrate-binding modules (CBMs) showed peaks at 90 m essentially anoxic zone. Clustering of CAZymes composition reflected heterogeneity in carbohydrate utilization by microorganisms with different lifestyles across depths (Fig. 8b). Intriguingly, MAGs harboring the WL pathway in completely anoxic zone encoded for a broader repertoire of CAZymes, such as *Desulfobacterota* MAGs had the broad cassette of GHs, combined with a high abundance of GHs in completely anoxic zone (Fig. 8c). It has been long believed that the WL pathway is used in diverse metabolic processes, and is most suitable for mixotrophy [72]. Mixotrophic microorganisms may use as few as two metabolic strategies simultaneously or switch between different strategies for carbon acquisition [74]. Microorganisms in the YBH anoxic zones primarily exhibited mixotrophic lifestyle, with a considerable diversity of energy metabolisms including central carbon metabolisms and fermentation. The central carbon metabolism pathways included glycolysis, TCA pathway and gluconeogenesis (Fig. 7, and Table S9). The majority of MAGs in the anoxic zones harbored a complete TCA pathway and utilized oxidative phosphorylation to produce ATP. NAD(P)H dehydrogenase (*ndh*) was strictly limited to photoautotrophic *Cyanobacteria* (bin193, bin65), whereas complex I (*nuo*) and complex II (*sdh*/*frd*) were widely distributed among all other MAGs (Table S10). The *ldhA* gene for metabolic transformation between lactate and pyruvate was found in seven MAGs assigned to *Gammaproteobacteria*, *Dehalococcoidales*, *Desulfobacterales*, *Planctomycetota*, and *Spirochaetia*. Only the *Gracilibacteria* MAGs harbored the genes for formate formation from pyruvate (formate C-acetyltransferase, *pflD*), whereas the majority of MAGs had the potential to oxidize formate to CO_2_ and H_2_ by possessing the *fdh*/*fdo* gene. The key genes of acetate metabolism were detected in MAGs mainly affiliated to *Desulfatiglandaceae*, *Desulfobacterales* and *Planctomycetota* (Fig. 7). Although these MAGs had uncomplete WL pathway, most members of acetogenic bacteria also showed an outstanding metabolic flexibility for utilizing a vast variety of different substrates for heterotrophy, including one-carbon compounds (formate, methanol, and methyl groups from many methoxylated aromatic compounds), two-carbon compounds (glyoxylate, glycolate, and oxalate), lactate, and pyruvate [72, 75]. In contrast to autotrophic growth, metabolic flexibility is seen as a key ability of acetogens to explain the almost-ubiquitous distribution of acetogenic bacteria in anoxic YBH. Thus, these mixotrophs with the WL pathway, could assimilate organic compounds in addition to the fixation of CO_2_, and had a competitive advantage over obligate autotrophs or heterotrophs in the deep YBH. Notably, there are examples of organisms with the presence of two different autotrophic pathways [76, 77], such as bin148 (*Bathyarchaeia*) not only encoded genes for the WL pathway, but also contained genes required for CO_2_ fixation via the CBB cycle (Fig. 3). This allowed us to speculate that conditional usage of different CO_2_ fixation pathways may be especially advantageous for those microorganisms living under extreme environments. In a high-energy situation, the organisms fixed CO_2_ via the CBB cycle or use it as an alternative biosynthesis pathway of ribulose-1,5-disphosphate in archaea that is generally deficient in phosphate dikinase, whereas under low-energy conditions, they switched to the energetically more favorable WL pathway. Additionally, members of the phylum *Chloroflexi* were active in both heterotrophic and autotrophic incubations [78], so it is possible that these organisms would have a mixotrophic strategy for carbon assimilation. These results indicated that the microbial community appeared to be predominantly mixotrophic with a highly flexible carbon acquisition strategy in the anoxic zones.

## Conclusion

Pathway-specific metagenomic analyses and reconstruction of individual genomes in the YBH demonstrated an explicit and complete redox partitioning of the carbon fixation pathways. These redox gradients favored growth of photoautotrophs, chemolithotrophs, and mixotrophs that use distinct autotrophic pathways spanning the oxic, hypoxic, essentially anoxic and completely anoxic zones. Photosynthesis-based CBB cycle play a quantitatively important role in oxic zone. Metabolically versatile microorganisms dominated key successional redox gradients and multiple types of energy generation in hypoxic zone and essentially anoxic zone. In the absence of light, the WL pathway has been highlighted as the additional metabolic and most energy-efficient pathways for autotrophic carbon fixation in anoxic zones. Functional predictions of the metabolic pathways in MAGs from the YBH anoxic zones provided our first glimpses into roles of the mixotrophic lifestyle of carbon fixation microorganisms in carbon cycling. Although we lacked experimental evidence that these genomes can indeed perform inorganic carbon fixation, our results revealed their potential genetic capacity and motivated future experiments to characterize the ecological relevance of novel chemolithoautotrophic and mixotrophic lineages in the YBH.

### Supplementary information

Supplementary figures

Supplementary tables

## Data Availability

The datasets generated for this study were deposited in the national omics data encyclopedia (NODE) under BioProject number OEP003486.
