# Supplementary material for: Phylogenetically and metabolically diverse autotrophs in the world’s deepest blue hole
Source: ISME Commun. 2023 Nov 14;3:117. doi: 10.1038/s43705-023-00327-4 (PMC10645885; doi:10.1038/s43705-023-00327-4)
Supplement: Supplementary file 1 — Supplementary figures [file 43705_2023_327_MOESM1_ESM.docx]

**Phylogenetically and metabolically diverse autotrophs in the world's deepest blue hole**

**Xing Chen^1+^, Jiwen Liu^1,2,3+^, Xiao-Yu Zhu^1^, Chun-Xu Xue^1^, Peng Yao^2,4^, Liang Fu^5^, Zuosheng Yang^6^, Kai Sun^1^, Min Yu^1,2,3^, Xiaolei Wang^1^, Xiao-Hua Zhang^1,2,3*^**

^1^ Frontiers Science Center for Deep Ocean Multispheres and Earth System, and College of Marine Life Sciences, Ocean University of China, Qingdao 266003, China

^2^Laboratory for Marine Ecology and Environmental Science, Laoshan Laboratory, Qingdao 266237, China

^3^Institute of Evolution & Marine Biodiversity, Ocean University of China, Qingdao 266003, China

^4^Key Laboratory of Marine Chemistry Theory and Technology, Ministry of Education, Ocean University of China, Qingdao 266100, China

^5^Sansha Track Ocean Coral Reef Conservation Research Institute, Sansha 573199, China

^6^College of Marine Geosciences, Ocean University of China, Qingdao 266100, China

***Author for correspondence:**

Xiao-Hua Zhang, College of Marine Life Sciences, Ocean University of China, 5 Yushan Road, Qingdao 266003, PR China, Tel/Fax: +86-532-82032767, Email: [xhzhang@ouc.edu.cn](mailto:xhzhang@ouc.edu.cn)

**Supplementary figures**


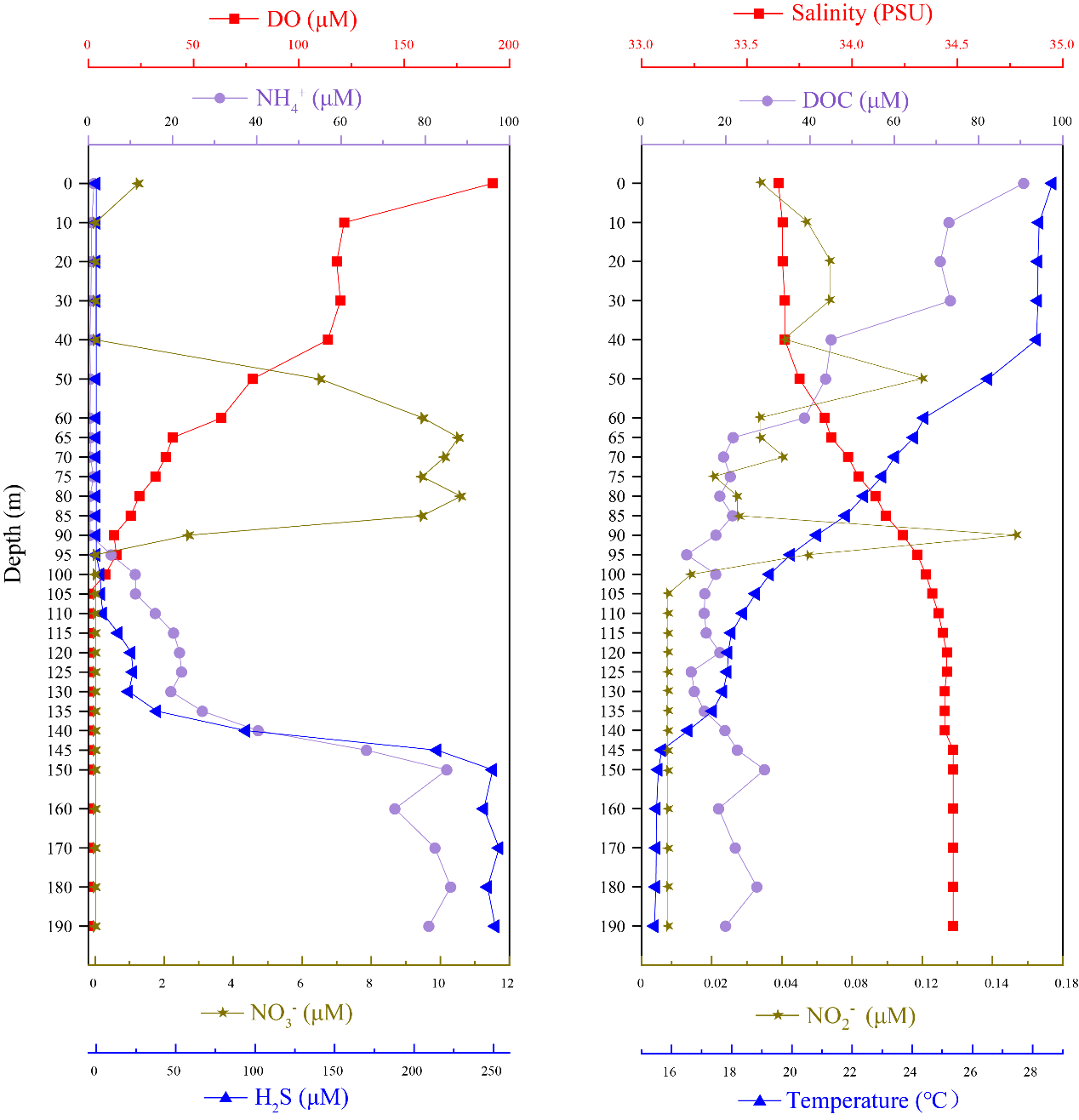


**Figure S1:** The Yongle blue hole water column in October 2019 was highly stratified with physicochemical differences.


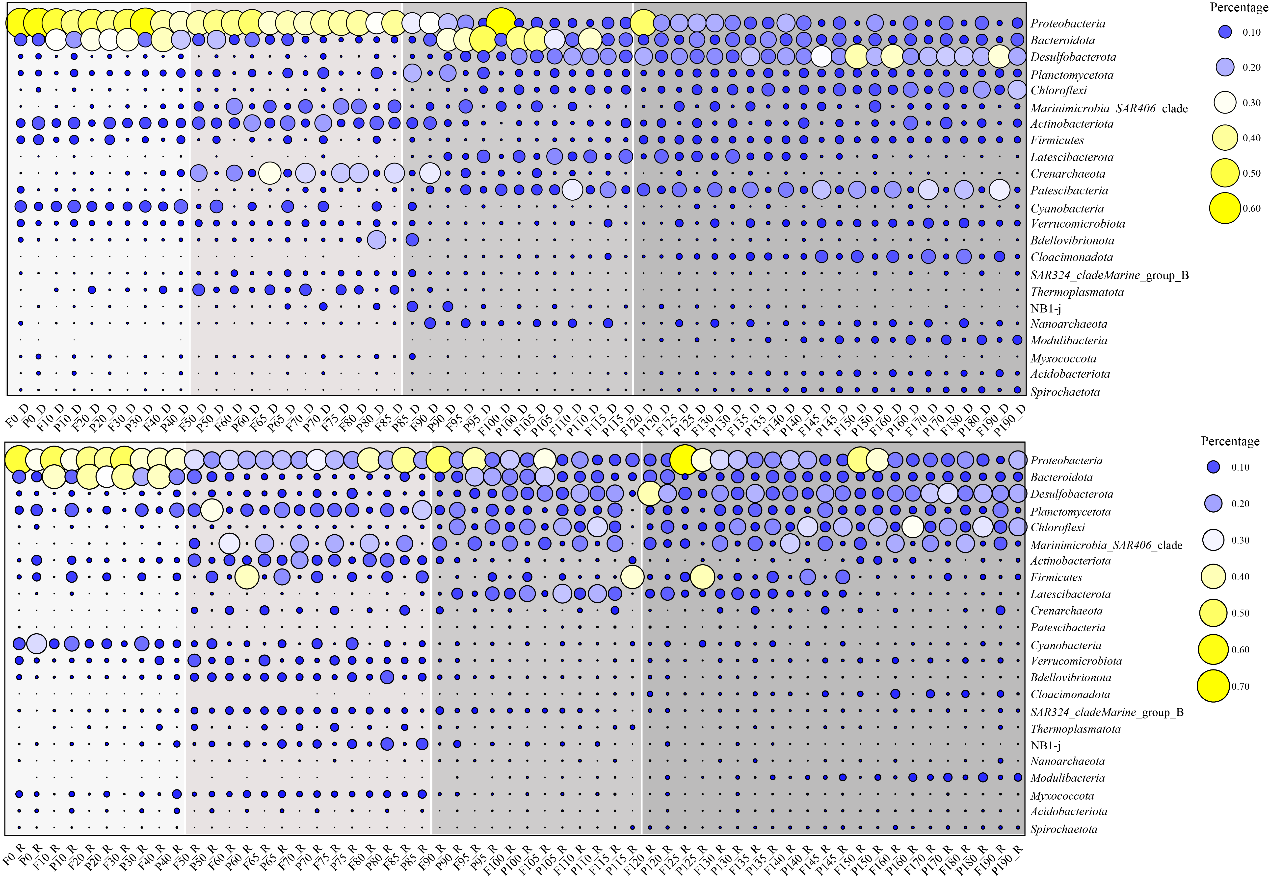


**Figure S2:** The relative abundance of microbial community present across the YBH at phylum level by DNA and RNA sequencing.


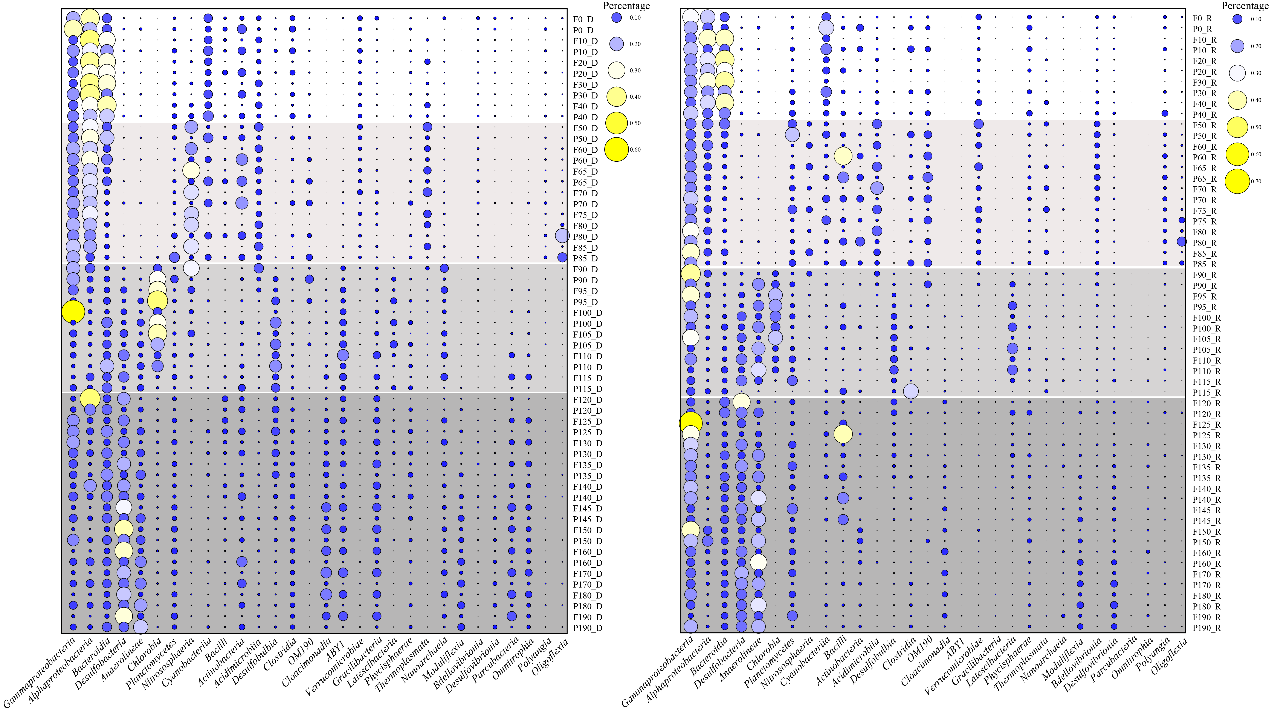


**Figure S3:** The relative abundance of the microbial community present across the YBH at the class level as defined by DNA and RNA sequencing.


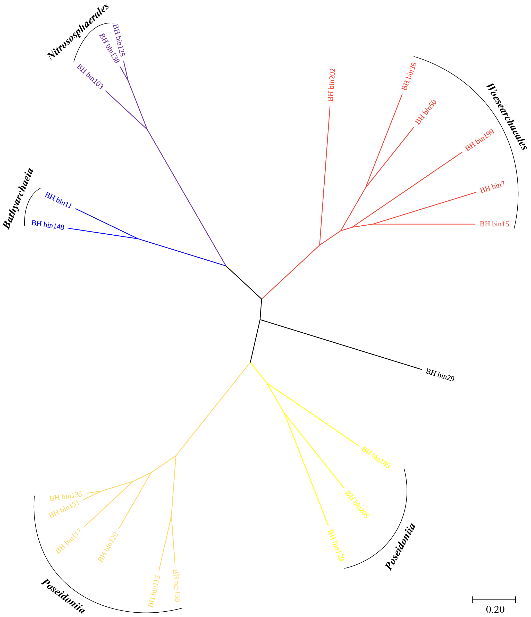


**Figure S4:** Phylogenetic analysis of 22 archaeal MAGs based on 122 single-copy protein-coding marker genes. MAGs were assigned to clusters represented by different branch colors. The scale bar represents 0.2 substitutions per site.


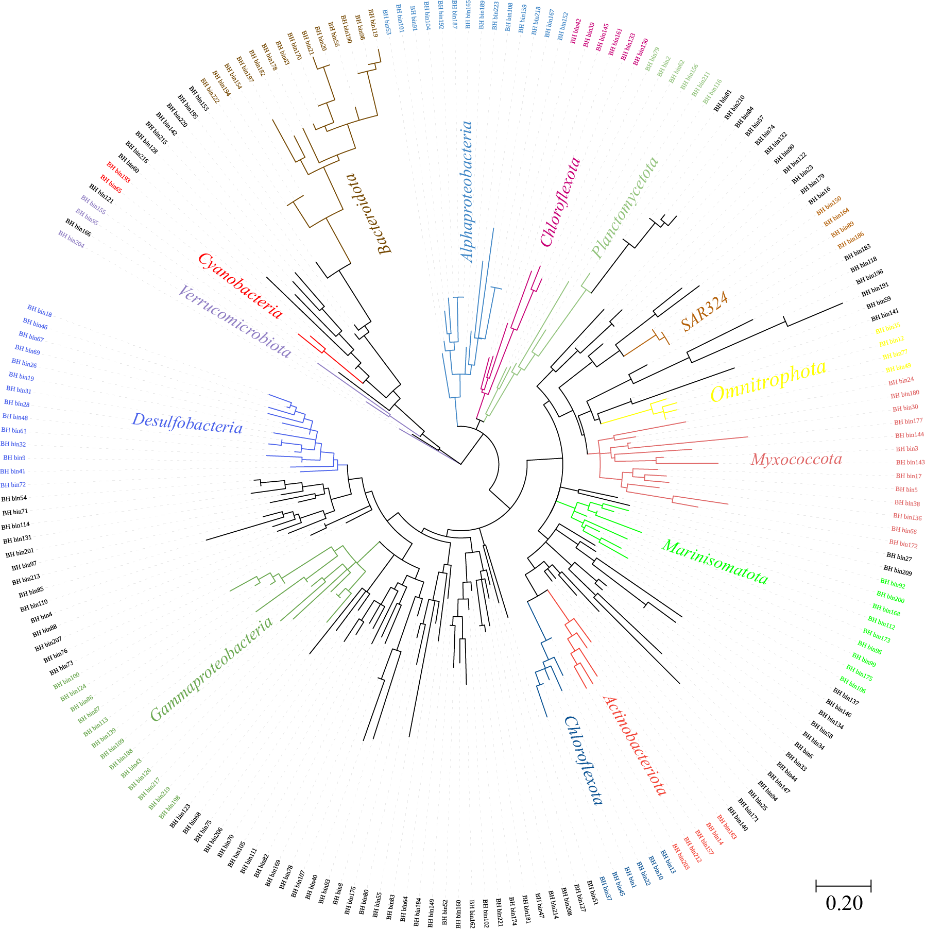


**Figure S5:** Phylogenetic analysis of 222 bacterial MAGs based on 120 single-copy protein-coding marker genes.


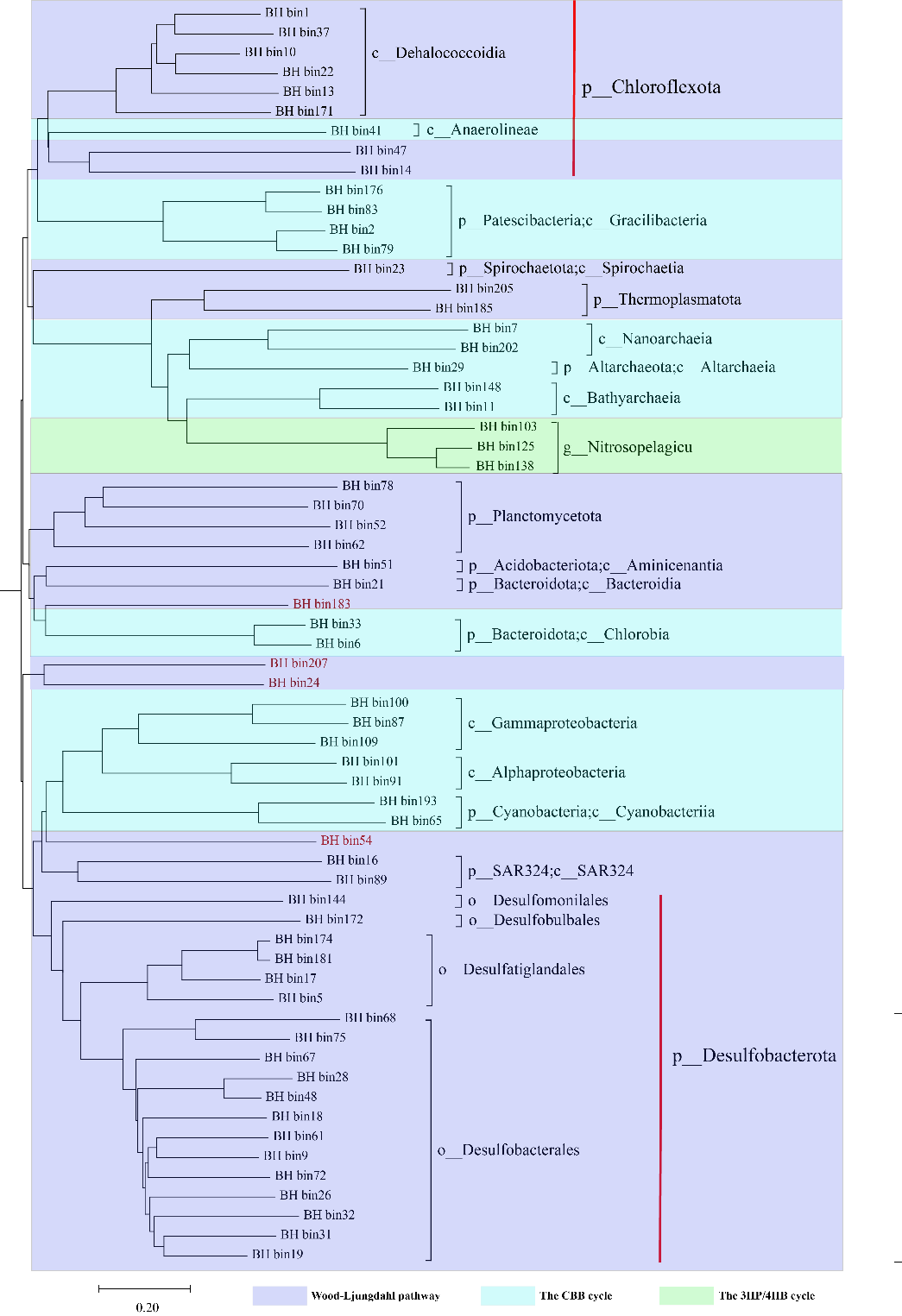


**Figure S6:** Phylogenetic analysis of 64 carbon fixation MAGs based on 16S rRNA sequences. Bootstrap calculated by maximum likelihood in IQ-Tree under the test option (100 replicates). MAGs were assigned to different pathways clusters. The scale bar represents 0.2 substitutions per site.
